# Behavioral practices towards antibiotic use among health care workers - Sierra Leone, 2021: a facility-based cross-sectional study

**DOI:** 10.11604/pamj.2024.47.63.39287

**Published:** 2024-02-12

**Authors:** Aminata Tigiedankay Koroma, Patrick Maada Bundu, Musa Sheriff, Brima Baryon, Brima Gamaga, Foday Sillah, Munis Lebbie, Daniel Ngobeh, Matilda Mattu Moiwo, Jefery Morrison, Abu Dim Din Sesay, Samba Kamara, Mustapha Jalloh, Haurace Nyandemoh, Momoh Massaquoi, Kadijatu Nabie Kamara, Joseph Sam Kanu, James Sylvester Squire, Jean Leonard Hakizimana, Adel Hussein Elduma, Gebrekrstos Negash Gebru

**Affiliations:** 1 Sierra Leone Field Epidemiology Training Program, Freetown, Sierra Leone,; 2 African Field Epidemiology Network, Field Epidemiology Training Program, Freetown, Sierra Leone,; 3 Department of Community Health, College of Medicine and Allied Health Sciences, University of Sierra Leone, Freetown, Sierra Leone; 4 National Disease Surveillance Program, Ministry of Health and Sanitation, Freetown, Sierra Leone,

**Keywords:** Antibiotics, antimicrobial resistance, knowledge, attitudes, practice

## Abstract

**Introduction:**

globally, antimicrobial resistance (AMR) kills around 1.27 million 700,000 people each year. In Sierra Leone, there is limited information on antibiotic use among healthcare workers (HCWs). We assessed antibiotic prescribing practices and associated factors among HCWs in Sierra Leone.

**Methods:**

we conducted a cross-sectional survey among HCWs. We collected data using a questionnaire containing a Likert scale for antibiotic prescribing practices. We categorized prescribing practices into good and poor practices. We calculated adjusted odds ratios (aOR) to identify risk factors.

**Results:**

out of 337 (100%) HCWs, 45% scored good practice. Out of the total, 131 (39%) of HCWS considered fever as an indication of antibiotic resistance and 280 (83%) HCWs prescribed antibiotics without performing microbiological tests and 114 (34%) prescribed a shorter course of antibiotics. Factors associated with good practice were being a doctor (aOR=1.95; CI: 1.07, 3.56), the internet as a source of information (aOR=2.00; CI: 1.10, 3.66), having a high perception that AMR is a problem in the health-facility (aOR=1.80; CI: 1.01, 3.23) and there is a connection between one´s prescription and AMR (aOR=2.15; CI: 1.07, 4.32).

**Conclusion:**

this study identified a low level of good practice toward antibiotic prescription. We initiated health education campaigns and recommended continuous professional development programs on antibiotic use.

## Introduction

Antimicrobial resistance (AMR) arises when resistant strains of microorganisms such as bacteria, fungi, viruses, and parasites emerge as a result of selective pressure when these microorganisms are exposed to antimicrobial agents [1]. Globally, it is projected that the annual deaths caused by AMR strains of bacteria, viruses, fungi, and protozoa will rise from the current 1.2 million deaths to 10 million deaths annually [2]. The emergence and proliferation of these resistant strains make antimicrobial agents non-effective, which may lead to treatment failure and high mortality rate [3]. Antimicrobial resistance (AMR) complicates infectious disease treatment, and can lead to the spread of these diseases, increasing the hospital stays and medical costs [4]. Although the emergence of resistant strains may occur naturally, it has been exacerbated by human actions such as irrational drug use, inadequate surveillance, poor Infection Prevention and Control (IPC) programs, weak laboratory capacity, and poor-quality medicines among others.

Healthcare workers play a key role in the antibiotic resistance particularly those who are involved in prescribing antibiotics in clinics and hospitals. Several studies were conducted to assess the knowledge and prescribing behavior. These studies have shown that antibiotic prescription is a vital problem in hospitals [5,6]. A study conducted in India among doctors indicated that AMR is not just a problem in their hospitals but in the entire country [7]. Also, a substantial proportion of clinicians failed to identify the correct choice of antibiotics in a case-based scenarios study [8]. Another study conducted in India found that knowledge did not influence practices as 77% of the respondents had higher knowledge, but 87% reported that they treat viral infections with antibiotics [9]. In Pakistan, physicians said that their decision to use antibiotics was influenced by patients´ demands, and the choice of the drug was determined by the socioeconomic status of the patients [10]. A study conducted among physicians in Nigeria in 2021, showed that 22% of the physicians had good knowledge, 40%, had a positive attitude, 32% had a good practice, and 32% had good prescribing practice of antibiotics [11].

In Sierra Leone, a study conducted in a tertiary hospital has shown that a high resistance rate of antibiotics was associated with the extended-spectrum beta-lactamase-producing organisms [12]. In different study conducted among medical doctors, it showed that the prescribing patterns of antibiotics were irrational [13]. Furthermore, another study indicated that 87% of prescription for children under five years old contained antibiotics [14]. Although some studies have been conducted on the perceptions of healthcare workers and drivers of antibiotics resistance, limited studies have been conducted among healthcare workers to assess knowledge, attitude, and prescribing practices on antibiotic use in Sierra Leone. It is essential to understand the awareness, prescribing behaviors, and practices of healthcare workers and dispensers regarding antibiotic use and resistance. Therefore, we assessed the healthcare workers knowledge, attitudes, and prescribing practices toward antibiotics use and identified associated factors in Sierra Leone. The findings of this survey will be used to develop and implement appropriate interventions to reduce AMR in the country. Also, it will inform policy-makers on effective AMR prevention and control strategies in the country.

## Methods

**Study design:** we conducted a facility-based cross-sectional study to assess the antibiotic prescribing practices and associated factors among healthcare workers working in selected healthcare facilities of Sierra Leone.

**Study setting:** Sierra Leone is located in West Africa, bordered by the North Atlantic Ocean, Guinea, and Liberia, and has a total surface area of 71,740 km^2^. The estimated population is 7,908,687 [15]. The country has five administrative regions namely: Eastern, Northern, Southern, North-West, and Western Area. The regions are divided into 16 districts with almost half of the population of Sierra Leone living in Freetown, the capital city. The country has a three-tier healthcare delivery system: tertiary, secondary, and primary levels. The tertiary level comprises of national and regional referral hospitals, the secondary level is comprised of district hospitals and the primary level contains Community Health Centers (CHC), Community Health Posts (CHP), and Maternal-Child Health Posts (MCHP). At the time of the survey, Sierra Leone had 1543 healthcare facilities including 85 hospitals, 265 CHCs, 200 clinics, 438 CHPs, and 666 MCHPs. The study was conducted from May to August 2021.

**Study population:** the study population involved healthcare workers working at various levels of the healthcare system. Healthcare workers in this study included medical doctors, community health officers, midwives, nurses, community health assistants, pharmacists, and pharmacy technicians. Any healthcare provider who prescribes or dispenses medications for at least six months in the selected healthcare facilities was included in the survey. Any healthcare provider who does not prescribe or dispense and has not worked in a health facility for more than six months was excluded from the study.

**Sampling size and sampling technique:** a total number of 337 healthcare workers were selected based on a 95% confidence level, 80% power, a proportion of 50%, and a margin of error of 5%. Ten percent of the calculated sample size was added to account for the non-response rate [16].

**Figure 1:**
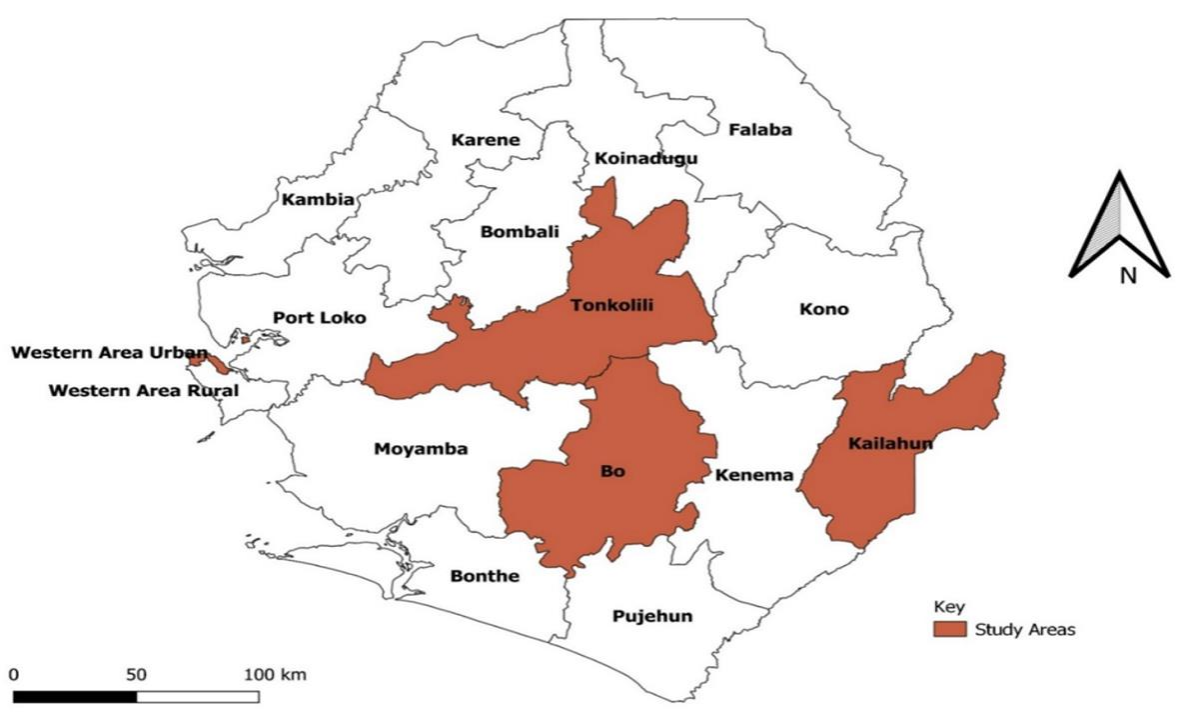
districts where health facilities and study participants were selected, Sierra Leone

**Selection of healthcare facilities:** we selected one referral hospital in Western Area Urban, five secondary-level hospitals, and one primary healthcare unit. In the Southern Region, we selected one referral (tertiary hospital), three secondary hospitals, and two primary healthcare units. In the Northern region, one referral hospital, two secondary hospitals, and two primary healthcare units were selected. In the Eastern region, we selected one referral hospital, two secondary hospitals, and two primary healthcare units (Figure 1).

**Selection of healthcare workers:** healthcare workers were stratified by cadres, and simple random sampling techniques using the staff list as a sampling frame were used to select the required number of the study participants. Proportional to size was used to allocate the number of participants by cadres in each selected health facility.

**Data collection:** a structured pre-tested questionnaire was developed to collect data from study participants. An electronic version of the questionnaire was created in Epi-Info 7. Data was collected using the Epi-Info APP installed on Android or IOS devices such as phones or tablets. The questionnaire was pre-tested by selecting 5% of health facilities in Western Area Rural, which is not part of this study. The questionnaire was updated based on the findings from the pilot test exercise. The updated questionnaire from the pretest was used as a final version for the data collection. Demographic variables such as age, sex, cadre, professional category, number of years at work (experience), and knowledge, perceptions, and practice-related information regarding antibiotic resistance were collected from healthcare workers or dispensers.

### Operational definition of terms

**Knowledge:** in this survey knowledge refers to the study participants´ (healthcare workers´ or dispensers´) understanding of any given topic, in this case, antimicrobial usage, and antimicrobial resistance.

**Attitude:** refers to healthcare workers feelings or perceptions towards this subject (antimicrobial usage and antimicrobial resistance), as well as any preconceived ideas that they may have towards it.

**Practice:** refers to the ways in which they demonstrate their knowledge and attitude through their actions. In this study, the actual practice of the respondents for the prevention of antimicrobial resistance was considered.

**Data analysis:** data was captured and analyzed using Epi Info version 7.2. Knowledge and attitude were assessed by a set of 20 and six questions respectively using a five-point Likert´s scale. The scoring system for knowledge and attitude was: 5=strongly agree with the statement, 4=agree, 3=neutral, 2=disagree and 1=strongly disagree. The average scores for both overall knowledge scores and overall attitude scores of participants were calculated as 67 and 25 respectively. Participants who had the score above the average score, which is above 67 and above 25 were considered as having good knowledge and positive attitude or high perception respectively. The participants who had a score of 67 and below and a score of 25 and below were considered as having poor knowledge and negative attitude or low perception respectively.

Practice was assessed by a set of 10 questions. Correct responses were scored as “1” while wrong or do not know the answer was scored as “0”. The overall practice score ranged from 0 to 10. The average of the overall practice scores of participants was set as a cut-off to classify those with scores above the average of 6 were considered as good prescribing practice and those whose score was equal or below 6 were considered as bad prescribing practice.

Frequencies and proportions for the various variables were calculated. The measures of association (odds ratio (OR) at 95% confidence interval (CI)) and measures of statistical significance (Chi-square analysis) were performed to test for significant associations among different dependent and independent variables. Statistical significance was determined based on p-value of < 0.05. Results were summarized and displayed using frequency tables, charts, and figures.

**Institutional review board statement:** the study was approved by the Sierra Leone Ethics and Scientific Review Committee (Approval Code: Version: 20 March 2020). Collected data were stored in a secure protected computer. This study was reviewed by the US Centers for Diseases Control and Prevention under the project determination policy and was conducted consistent with applicable federal law and CDC policy.

**Informed consent statement:** written informed consent was obtained from participants. Confidentiality of participants was maintained and no personally identifiable information will be disclosed in any presentation or publication.

## Results

**Sociodemographic characteristics of healthcare workers:** a total of 337 healthcare workers were included in this survey with a response rate of 100%. Females accounted for 170 (50%) with median age of 34 years (20 years - 83 years). The majority of respondents, 143 (42%) were within the age group of 30 to 39 years, and 90 (27%) were within the age group 20 to 29 years. Healthcare workers were distributed across different level of cadre, the highest proportion, 95 (28%) was observed among nurses, followed by 89 (26%) among Community Health Officers (CHOs), and 84 (25%) among doctors. Most of participants, 332 (99%) had completed tertiary education and 293 (89%) had practiced for over two years. A total of 143 (42%) respondents were from the Western Area Urban. The highest percentage of healthcare workers, 250 (74%) were from those who working in hospitals. The majority, 286 (85%) of the health facilities included in this study were government-owned facilities.

**Resources on antimicrobial resistance at health facilities:** a total of 246 (73%) healthcare workers in this survey reported the availability of standard treatment guidelines in their health facilities and 302 (90%) indicated that they have a storage facility for drugs. Out of 336 healthcare workers interviewed, 268 (80%) did not receive any training on AMR in the past one year. Less than a half of healthcare workers, 146 (43%), indicated that they learnt about AMR during their academic training, and 143 (42%) did not learn about AMR in the past one year. Most of healthcare workers, 287 (85%), preferred senior colleagues as sources of specific information on AMR, whilst 262 (78%) indicated that they obtained information on AMR from the internet. A total of 189 (56%) healthcare workers reported having enough source of information when needed.

**Figure 2:**
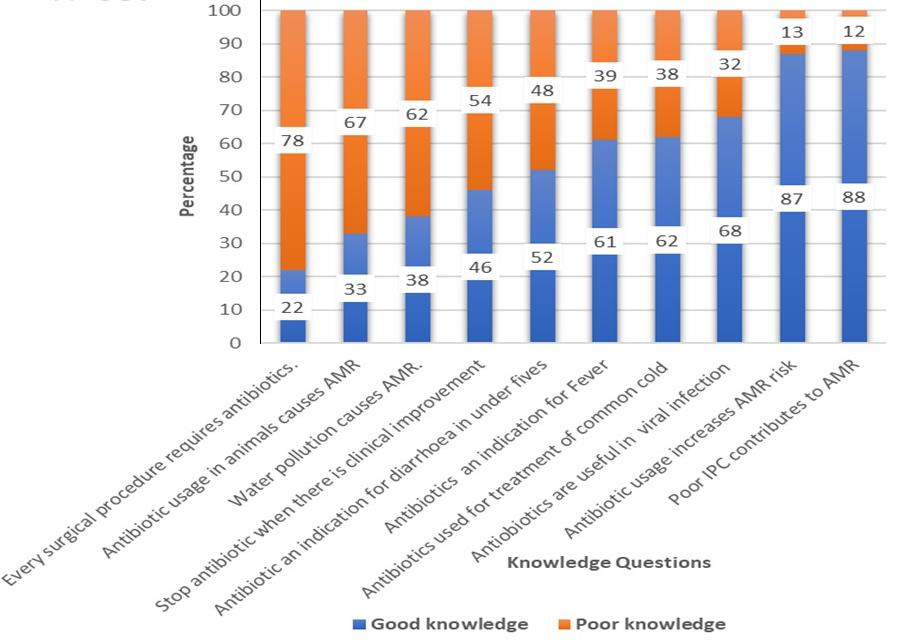
knowledge of the healthcare workers on antibiotic use and antimicrobial resistance, Sierra Leone, 2021 (N-337)

**Knowledge of healthcare workers on antibiotic use and antimicrobial resistance:** a total of 297 (88%) healthcare workers strongly agreed or agreed that poor infection prevention and control in health facilities contribute to the antimicrobial resistance and 253 (75%) of them strongly agreed or agreed that poor hand washing culture in healthcare settings contribute to antimicrobial resistance. The current study also revealed that 293 (87%) of respondents strongly agreed or agreed that the more antibiotics used in society, the higher the risk that resistance may develop and spread. A high proportion of respondents, 313 (93%), strongly agreed or agreed that patients should complete the full course of antibiotic treatment even when there is a clinical improvement, to reduce the risk of resistance. However, around half of the participants, 172 (51%) strongly agreed or agreed that antibiotics should be stopped immediately when the patient is clinically improved to reduce the risk of resistance. Nearly, 243 (72%) of participants wrongly knew (strongly agreed or agreed) that antibiotics are indicated after every surgical procedure to avoid complications and 41% incorrectly strongly agreed that antibiotics are indicated in most cases of diarrhea in children under five years of age. Of study participants, 111 (33%) strongly agreed or agreed that antibiotic use for animals can reduce the possibility of effective antibiotic treatment for humans (Figure 2).

**Level of knowledge by healthcare workers´ cadre:** the overall score of good knowledge was 51%. The highest proportion of good knowledge was among doctors, 84 (89%), followed by pharmacist 12 (58%), the CHOs, 89 (54%), midwife 37 (41%), nurses, 95 (27%), and community health assistant 15 (13%).

**Figure 3:**
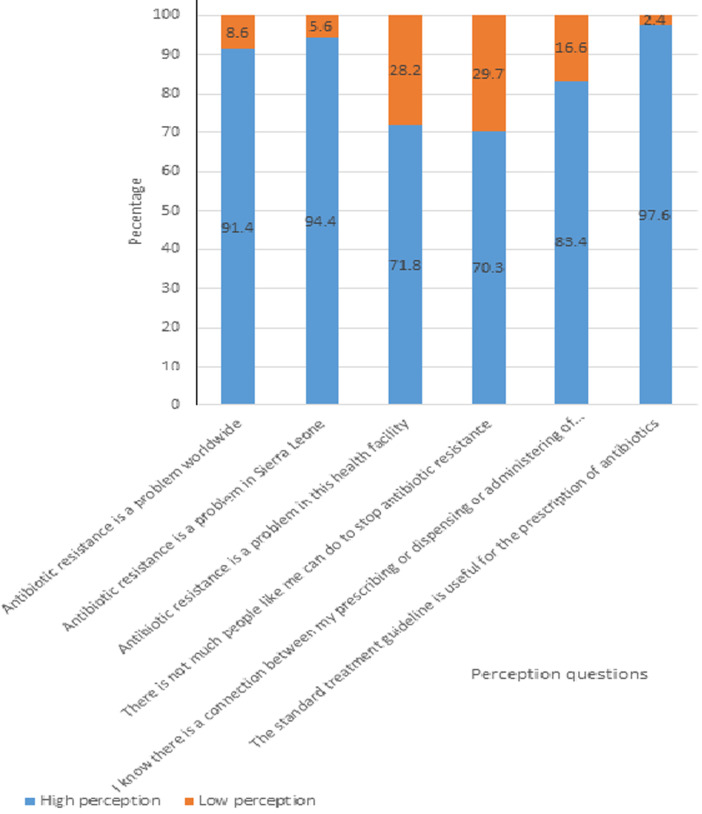
perception of the healthcare workers on antibiotic use and antimicrobial resistance, Sierra Leone, 2021 (N-337)

**Perception of healthcare workers on antibiotic use and antimicrobial resistance:** of the 337 respondents, 167 (50%) had an overall positive perception towards antibiotic use and antibiotic resistance. A total of 308 (91%) of the healthcare workers, 318 (94%) strongly agreed, and 242 (72%) agreed that antibiotic resistance is a problem worldwide, in Sierra Leone, and in their health facilities, respectively. Two hundred thirty-seven (70%) respondents strongly disagreed or disagreed that there are not much people like them can do to stop antibiotic resistance. A total of 281 (83%) health care workers knew that there is a connection between their antibiotic prescribing or dispensing behavior and the emergence and spread of antibiotic-resistant bacteria. The majority of respondents, 329 (98%) strongly agreed or agreed that standard treatment guideline is useful for the prescription of antibiotic (Figure 3).

**Practice of healthcare workers on antibiotic use and antimicrobial resistance:** the overall good practice of the healthcare workers included in this study was 45%. Out of 337 respondents, 132 (39%) accepted that they prescribed an antibiotic when they should not have. A total of 188 (56%) healthcare workers reported that they prescribed antibiotics out of fear of patient deterioration. Our result indicated that 60 (18%) respondents accepted that they prescribed antibiotics because it takes less time than to explain the reason why they are not indicated. One hundred eighty (53%) healthcare workers accepted that they had prescribed an antibiotic in a situation in which they couldn't conduct a follow-up of the patient. Fifty-two (15%) of healthcare workers said that they prescribed antibiotics to maintain a relationship with the patients. A total of 171 (51%) respondents replied that they prescribed antibiotics because they were uncertain of the diagnosis. Almost, 108 (32%) respondents reported that they prescribed a shorter course of antibiotic treatment as compared to available guidelines. Totally, 276 (82%) respondents replied that they prescribed antibiotics without carrying out microbiological tests. Finally, 198 (59%) healthcare workers said that they discontinued treatment earlier because the bacterial infection was not likely.

**Multivariate logistic regression analysis for factors associated with good practice towards antibiotic use:** all variables that were found to be associated with good practice in bivariate analysis were put into multivariate logistic regression to control confounding factors. Being a doctor as designation compared to other cadres (aOR=1.95, 95% CI: 1.07, 3.56; p-value = 0.028); having the internet as source of information on antibiotic use and on AMR (aOR= 2.00, 95% CI: 1.10, 3.66; p-value=0.0024); having a positive perception that antibiotic resistance is a problem in the health facility (aOR=1.80, 95% C.I: 1.01 , 3.23; p-value= 0.045), and having positive perception that there is connection between one´s antibiotic prescribing behavior and emergence and spread of antibiotic-resistant bacteria(aOR =2.15, 95% CI: 1.07, 4.32; p-value= 0.032), were factors statistically associated with good practice on antibiotic use (Table 1).

**Table 1:** bivariate and multivariate logistic regression analysis for factors associated with good prescribing practice, Sierra Leone, 2021

Variable	Practice		cOR	95% CI	aOR (95% CI)	p-value
	**Good**	**Poor**				
**Age group**						
20-35 years	98	97	1.6	1.03 - 2.48	1.5 (0.94-2.43)	0.085
>35 years	55	87				
**Sex**						
Male	89	78	1.9	1.223-2.917	1.4 (0.82-2.28)	0.226
Female	64	106				
**Designation**						
Doctors	53	31	2.6	1.571-4.355	1.9 (1.07-3.56)	0.028
Other health care workers	100	153				
**Overall perception on antibiotic use and AMR**						
High	89	78	1.9	1.224- 2.918	1.1 (0.65-1.89)	0.709
Low	64	106				
**Overall knowledge on antibiotic use and AMR**						
Good	88	85	0.6	1.0235- 2.429	0.7 (0.3 - 1.29)	0.256
Poor	65	99				
**Don’t learn about AMR**						
Yes	55	88	0.6	0.394 - 0.949	0.7 (0.44-1.15)	0.163
No	98	96				
**Use internet as source of information on AMR**						
Yes	132	130	2.6	1.492-4.567	2.0(1.10-3.66)	0.024
No	21	54				
**Antibiotic can be used to treat infections caused by fungi**						
Correct answer	104	105	1.6	1.02-2.50	1.5 (0.64-2.09)	0.637
Incorrect answer	49	79				
**Antibiotic resistance is a problem in this health facility**						
High perception	124	118	2.4	1.44-3.96	1.8 (1.01-3.23)	0.045)
Low perception	29	66				
**I know there is a connection between my prescribing or dispensing or administering of antibiotics and the emergence and spread of antibiotic-resistant bacteria**						
High perception	137	144	2.4	1.27-4.44	2.2 (1.07-4.32)	0.032
Low perception	16	40				

AMR: antimicrobial resistance

## Discussion

Antimicrobial resistance is a global health problem and development threat that needs an urgent multi-sectoral action. According to the WHO, AMR is one of the top 10 global public health threats facing humanity [17]. The aim of this study was to assess the knowledge, attitude, and practice towards antibiotic use among healthcare workers in Sierra Leone, as well as to determine factors associated with good practice of antibiotic use. Our findings indicated that 51%, 49%, and 45% of healthcare workers had respectively good knowledge, positive attitude, and good prescribing practice of antibiotics. These findings indicated that the study participants had good knowledge, practices, and high perceptions towards antibiotic use and antimicrobial resistance in compared with a study conducted in Nigeria where participants had lower levels of knowledge and practices, and low perceptions [11]. The knowledge of healthcare workers in our study was lower than the knowledge of healthcare workers in Egypt and Nigeria [18,19]. These differences can be attributed to the methodology used to score knowledge, attitudes, and practice or differences in the study subjects.

Our study showed that good knowledge was higher among doctors compared with other healthcare workers. This finding is similar to a result of a national survey conducted in Nigeria on AMR awareness and antibiotic prescribing behavior among healthcare workers, where physicians had better knowledge than other healthcare workers [20]. The highest level of knowledge among medical doctors was probably because they are more aware of the relationship between antibiotic use and resistance or they may have more access to guidelines and other learning platforms than others.

Our findings showed that a high percentage of standard treatment guidelines were available at their facilities. However, a low percentage of those who received training on AMR was found. These findings disagree in line with a study conducted in Egypt, where participants received more training and educational courses on antibiotic prescription [11,18]. Only one-fifth of the healthcare workers in our study had received training on AMR and half of them didn´t use available platforms to learn about AMR. This low level of training could be explained by the low proportion of respondents with good knowledge, higher perception towards AMR, and good prescribing practice. Lack of proper training and education is one of the most important factors increasing AMR in low and middle-income countries [21].

To reduce the risk of antibiotic resistance, the majority of our participants agreed or strongly agreed that patients should complete the full course of antibiotic treatment even if there is a clinical improvement. This result is in line with the findings of another study conducted in Nigeria, where 85% of participants knew that antibiotics should only be stopped after the completion of recommended doses. The majority of our study participants perceived that antibiotic resistance is a real problem in their health facilities. The same findings were shown in a study conducted in South Africa where all study participants believed that antibiotic resistance is a problem in their health facilities [22]. Studies conducted in Ghana and Nigeria found that the majority of participants agreed that AMR is a serious public health problem in their countries and around the world, and possibly a problem in their hospitals [17,22]. Other studies also found that large numbers of healthcare workers considered antimicrobial resistance to be a significant problem in their health facilities [11,23].

The majority of study participants agreed or strongly agreed that there is a connection between their prescribing behaviors and the emergency and spread of antibiotic resistance. This high perception may influence prescribing practice and reduce the emergency and spread of antibiotic-resistant pathogens. Our finding is consistent with a result of the study conducted in Nigeria, which showed that the majority of the participants did not implement an antimicrobial stewardship strategy which may contribute to antimicrobial resistance [11].

Our finding indicated that irrationality or misuse of antibiotics was high, which may contribute in the development of antibiotic resistance. This finding is similar to a study conducted in South Africa, which showed that 66% of healthcare workers were affected by the pressure from patients to prescribe antibiotics. A study conducted in Pakistan among physicians reported that their decision to use antimicrobials was influenced by patients´, demand and the choice of antibiotics determined by the socioeconomic status of the patient [24]. Several studies reported that bad prescribing practices are one of the main predictors of AMR, showing that excessive and inappropriate antibiotics prescribing is a driver of AMR [24,25]. Published literature has shown a positive correlation between increased antimicrobial use and the development of antimicrobial resistance [26].

Our study found that doctors had higher good practices compared to other healthcare workers. A similar finding was found in a study conducted in India, where nurses, pharmacy shopkeepers, and informal providers were more likely to perform poorly compared to allopathic doctors [9]. The significant association between designation and good practice can be explained by the fact that doctors were found to have better knowledge than other health workers. Our study found a significant association between the perception that AMR is a problem in the health facility and good practice. Also, we found a connection between one´s prescription and good prescribing practice. This finding is supported by studies showing that perceptions is influenced by health-related knowledge and motivator for appropriate antimicrobial use [18].

The use of the Internet as a source of information was also significantly associated with good prescribing practice. The Internet has become the most common source of health-related information and can be used to change health care workers´ behavior and promote health care workers´ good practices. Various studies have found the Internet to be one of the most useful sources of information to promote rational use of antibiotics [27,28]. Access to information sources can help healthcare workers to have good antibiotic-prescribing practices. It is not possible in a cross-sectional study to establish a temporal relationship between explanatory variables and the outcome because these are simultaneously assessed and it will be difficult to make causal inferences. We might not include other antibiotic prescribers such as traditional healers, which may create selection bias.

## Conclusion

This study provides information on the knowledge, attitude, and practice towards antibiotic use and AMR, as well as factors associated with good prescribing practices among healthcare workers in Sierra Leone. This study reported poor practices, slightly high knowledge, and low perception on antibiotics and AMR use. Factors associated with good prescribing practices included; designation, internet use as a source of information, perception that AMR is a problem in the health facility, and perception that there is a connection between one´s prescription and emergency and spread. We recommend that the Ministry of Health and Sanitation to develop a comprehensive strategy for continuous development programs on antibiotic use, and develop an antibiotic use enforcement strategy including monitoring, and supervision. We recommend HCWs to comply with good antibiotics prescribing practices. Also, health facility authorities to avail evidence-based sources of information platforms on antibiotics use including internet facilities.

### What is known about this topic

- *Antimicrobial resistance is a significant global public health problem;*
- *Irrational use of antibiotics produces pathogen resistance*.

### What this study adds

- *The study shows poor antibiotics use practices among healthcare workers;*
- *The study reported slightly high knowledge and low perception regarding antibiotics use among healthcare workers*.
